# Characterization of primary afferent spinal innervation of mouse uterus

**DOI:** 10.3389/fnins.2014.00202

**Published:** 2014-07-28

**Authors:** Geraldine Herweijer, Melinda Kyloh, Elizabeth A. H. Beckett, Kelsi N. Dodds, Nick J. Spencer

**Affiliations:** ^1^Discipline of Human Physiology and Centre for Neuroscience, Flinders UniversityAdelaide, SA, Australia; ^2^Discipline of Physiology, School of Medical Sciences, University of AdelaideAdelaide, SA, Australia

**Keywords:** uterus, pain, dorsal root ganglion, CGRP, spinal afferent

## Abstract

The primary afferent innervation of the uterus is incompletely understood. The aim of this study was to identify the location and characteristics of primary afferent neurons that innervate the uterine horn of mice and correlate the different morphological types of putative primary afferent nerve endings, immunoreactive to the sensory marker, calcitonin gene related peptide (CGRP). Using retrograde tracing, injection of 5–10 μL of 1,1′-didodecyl-3,3,3,3′-tetramethylindocarbocyanine perchlorate (DiI) into discrete single sites in each uterine horn revealed a biomodal distribution of sensory neurons in dorsal root ganglia (DRG) with peak labeling occurring between T13-L3 and a second smaller peak between L6-S1. The mean cross sectional area of labeled cells was 463 μm^2^ ± s.e.m. A significantly greater proportion of labeled neurons consisted of small cell bodies (<300 μm^2^) in the sacral spinal cord (S2) compared with peak labeling at the lumbar (L2) region. In both sections and whole mount preparations, immunohistochemical staining for CGRP revealed substantial innervation of the uterus by CGRP-positive nerve fibers located primarily at the border between the circular and longitudinal muscle layers (*N* = 4). The nerve endings were classified into three distinct types: “single,” “branching,” or “complex,” that often aligned preferentially in either the circular or longitudinal axis of the smooth muscles. Complex endings were often associated with mesenteric vessels. We have identified that the cell bodies of primary afferent neurons innervating the mouse uterus lie primarily in DRG at L2 and S1 spinal levels. Also, the greatest density of CGRP immunoreactivity lies within the myometrium, with at least three different morphological types of nerve endings identified. These findings will facilitate further investigations into the mechanisms underlying sensory transduction in mouse uterus.

## Introduction

The perception of pain from internal organs is a fundamental physiological phenomenon, with pain from the viscera one of most common symptoms in a variety of disease states (Ness and Gebhart, 1990; Robinson et al., 2004). The non-specific nature of visceral pain, especially in chronic disease, not only leads to a difficultly in diagnosis but also in effective, targeted treatment options (Cervero and Laird, 1999). Extensive studies of the colon and other visceral organs seek to address some of these issues (Ness and Gebhart, 1990), however the mechanisms underlying the detection and transmission of nociceptive signals from the lower pelvic regions including the female reproductive tract are far from understood (Stratton and Berkley, 2010; Chaban, 2012). The non-specificity of visceral pain is evident in patients with a variety of different conditions such as; endometriosis, irritable bowel disease and vulvodynia (Stratton and Berkley, 2010).

Sensory information from the uterus and other areas of the reproductive tract such as the cervix and vagina is conveyed to the central nervous system mainly via the pelvic nerves arising from the lumbosacral dorsal root ganglia (DRG). Additional inputs from the hypogastric and vagal nerves also transmit sensation centrally, arising rostrally via the T13-L4 region and directly from the brainstem via the nodose ganglia respectively (Inyama et al., 1986; Berkley et al., 1988; Collins et al., 1999). These relay sensory signals such as the degree of distension, nociception, and vascular state of the uterus and reproductive tract (Chaban, 2012). While the autonomic innervation and endocrine control of the uterus has been reasonably well characterized, in both health and disease states (Ness and Gebhart, 1990; Gnanamanickan and Llewellyn-Smith, 2011) few studies exist in mice; and no studies have characterized the primary afferent innervation in mouse uterus.

The cell bodies of afferent fibers carrying sensory signals from the periphery and viscera to the spinal cord are located in the dorsal root ganglia (DRG). Thus DRG allow for isolated study of sensory neurones, using both retrograde and anterograde tracing studies, which can ultimately provide a basis for further studies and insight into potential targets for effective and more specific management of visceral neuropathic pain conditions.

The aim of this study was two-fold. Firstly, to identify the location and characteristics of the cell bodies of primary afferent neurones that innervate the mouse uterine horn and secondly characterize the different types of CGRP immunoreactive primary afferent nerve endings innervating this region, including both in their localization within the uterine wall and morphological specializations.

## Materials and methods

### DiI retrograde labeling, DRG dissection and tissue preparation

**Figure 1:**
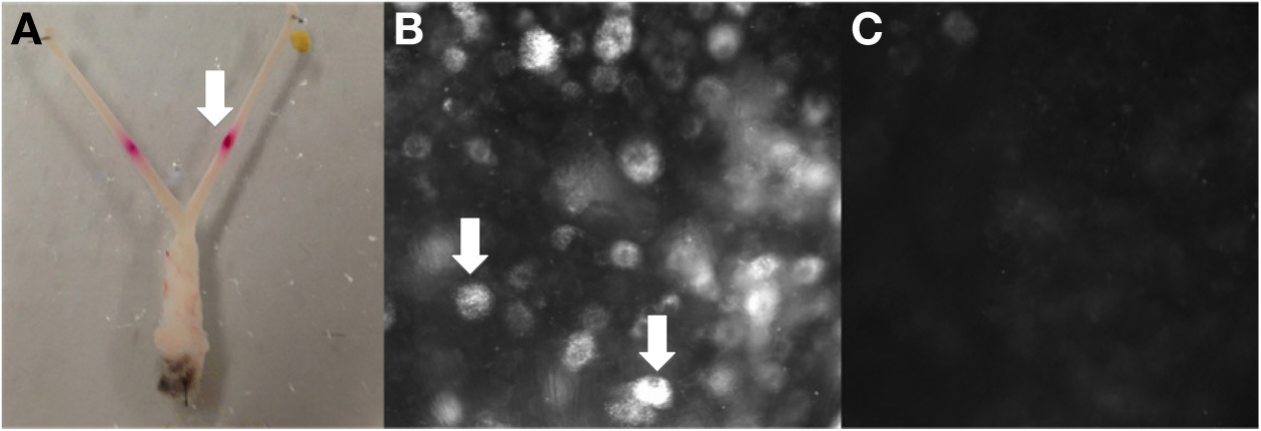
**Example of retrograde tracing observed in DRG after DiI injection into the uterus *in vivo*. (A)** Site of bilateral DiI injections into each uterine horn, **(B)** DiI labeled neurons (indicated by arrows (L2) and **(C)** no labeling (T6).

Four adult virgin female C57BL/J6 mice were used for surgical injections of the fluorescent neuronal tracer DiI (3,3′-didodecyl 1,1,1′,1′-tetramethylindocarbocyanine diluted with 1% dimethylformamide). The animals were anesthetized with 2–3% isofluorane inhalation, the depth monitored by an absence of any response to tail or hind limb pinch. Upon failure to elicit a tail or hind limb reflex, a midline laparotomy was made to expose the abdominal cavity. All experiments in this proposal were approved by the Animal Welfare Committee of Flinders University of South Australia (Ethics # 861/13). Internal organs that obstructed the uterus were reflected to expose both uterine horns. Approximately 5–10 μl of retrograde neuronal tracer DiI was injected bilaterally into the wall of each uterine horn, using a 25-gauge needle. The viscera was inspected for any leakage to surrounding organs, rinsed with sterile saline, then repositioned back into the abdominal cavity. The abdominal muscles and skin was closed with sutures and staples respectively and the animals were allowed to recover under constant observation. After 7 days postoperatively, the animals were euthanized by inhalation anesthetic. The uterus was removed and fixed in paraformaldehyde (4%) solution, with each horn cut longitudinally, stretched and pinned to the base of a Sylgard-lined organ bath. Other viscera organs were inspected for any residual, leaked tracer dye and it was found that the DiI was confined to the uterine wall (Figure 1A). The DRG from bilateral spinal level T1-S1 inclusive were removed with any excess connective tissue and spinal roots dissected before mounting on slides.

### Cell counting and cell size analysis

Whole mount preparations of each uterine horn were analyzed using an Olympus AX70 fluorescent microscope and CY3 fluorescence. Photographs of the DRG in the focal plane with peak labeled cells in focus were taken at 350 ms using the Analysis Olympus Version 5. DiI labeled neurons were distinguished from non-labeled cells by a distinct punctuated fluorescence in their cell bodies.

### Tissue preparation and immunohistochemistry of uterus

Four adult female virgin C57BL/6 mice were euthanized by inhalation anesthetic and both uterine horns from each mouse were removed. One uterine horn of each animal was cut longitudinally, along antimesenteric border, stretched and pinned in a Petri dish. The tissue was fixed in paraformaldehyde (4%) for one to two days at room temperature. After fixing, the full-thickness whole mounts were processed for immunohistochemistry. The tissue was washed and blocked for 1 h (1% BSA 5%NDS 1% triton) and incubated in primary antibody (CGRP Rabbit Peninsula A11729 concentration 1:3000) for 48 h. After primary incubation, the tissue was washed again and incubated in secondary antibody (DaR CY3 Jackson 105748) at 1:200 for 24 h. The tissue was washed, ragged edges trimmed and each horn was cut transversely into ovarian and cervical regions and mounted mesenteric side up on slides.

The other uterine horn was left intact, stretched and pinned in a petri dish. The tissue was fixed in paraformaldehyde (4%) for one day before cryo-protection for 12 h. Four 4–5 mm pieces of tissue were cut from equal distances along the horn and embedded in OCT medium and allowed to set at −20C for 1 h. Sections were cut on a cryostat (12–25 μm thick) and mounted sequentially on coated slides. Sections (1, 6, and 11) were retained from each animal for immunohistochemistry. After blocking slides (1% BSA 5%NDS 1% triton) for 1 h, the sections were incubated in primary antibody (CGRP Rabbit Peninsula A11729 concentration 1:1600) for 48 h. After primary incubation, the tissue was washed and incubated in secondary antibody DaR CY3 Jackson 105748 1:200) for 24 h. The slides were analyzed using an Olympus AX70 fluorescent microscope and CY3 fluorescence, photographs of were taken at 50–150 ms using the program Analysis Olympus Version 5.

## Results

### DiI labeled dorsal root ganglia: numbers and spinal level

Approximately 7 days after bilateral injections of DiI into each uterine horn (Figure 1A), whole-mount DRG preparations from four mice revealed cell bodies with distinct punctate fluorescence, characteristic of DiI labeling (Figure 1B). The number of DiI labeled cells varied between levels, whereby some DRG contained many neurons, such as those found at L2 and others such as in the upper thoracic DRG that contained no positively labeled neurons (Figure 1C). The distinct pattern of labeling at specific DRG spinal segments was consistent between animals, indicating that retrograde labeling was indeed specific from the injection sites in each uterine horn. This is supported by our post-mortem examination of the peritoneal cavity which revealed no leakage to other organs.

**Figure 2:**
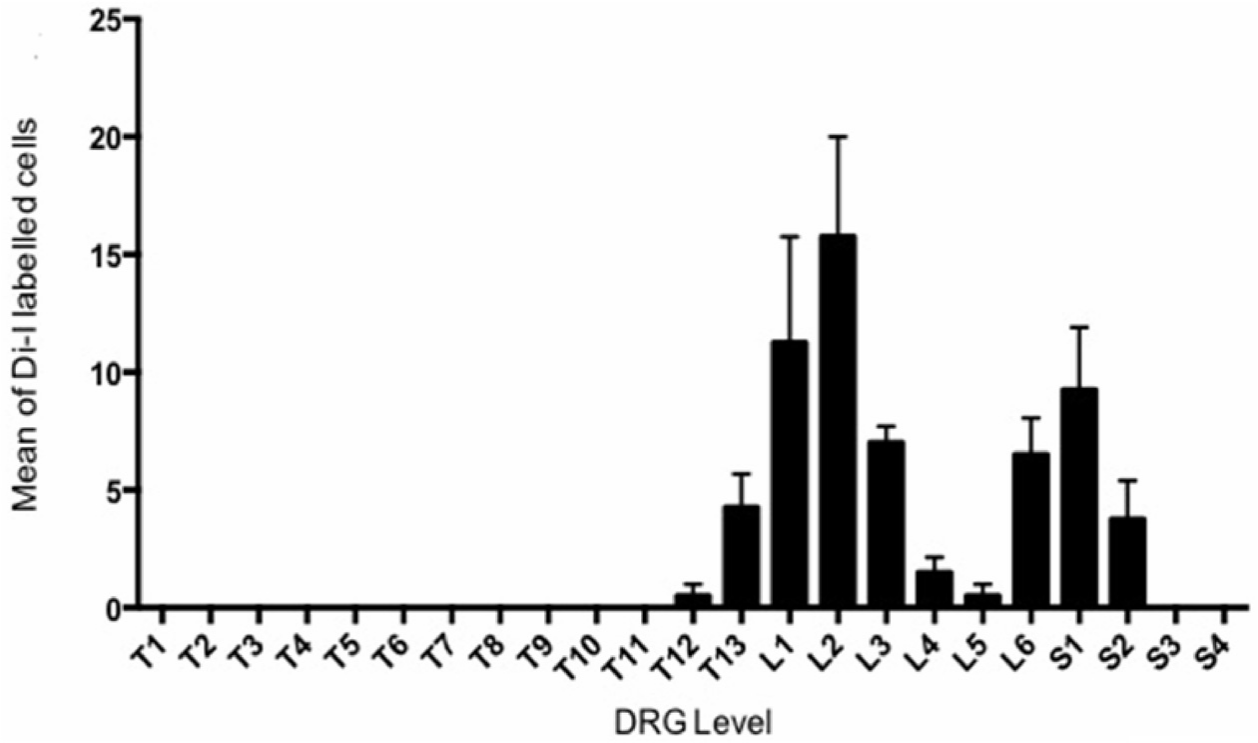
**Retrograde DiI labeling from bilateral injections into each uterine horns revealed to distinct peaks of sensory innervation in Dorsal Root Gangli**. The mean number of DiI labeled cells is plotted against the spinal DRG level in which they were observed. The counts of four animals were averaged at each level.

In total, 269 DiI labeled cells were counted from four mice. The distribution of the mean number of DiI labeled cell bodies for each animal counted at every DRG level (Figure 2) revealed two distinct peaks, one major peak spanning from T13 to L3 and a smaller, narrower peak from L6 to S2.

### DiI labeled dorsal root ganglia: cell size

**Figure 3:**
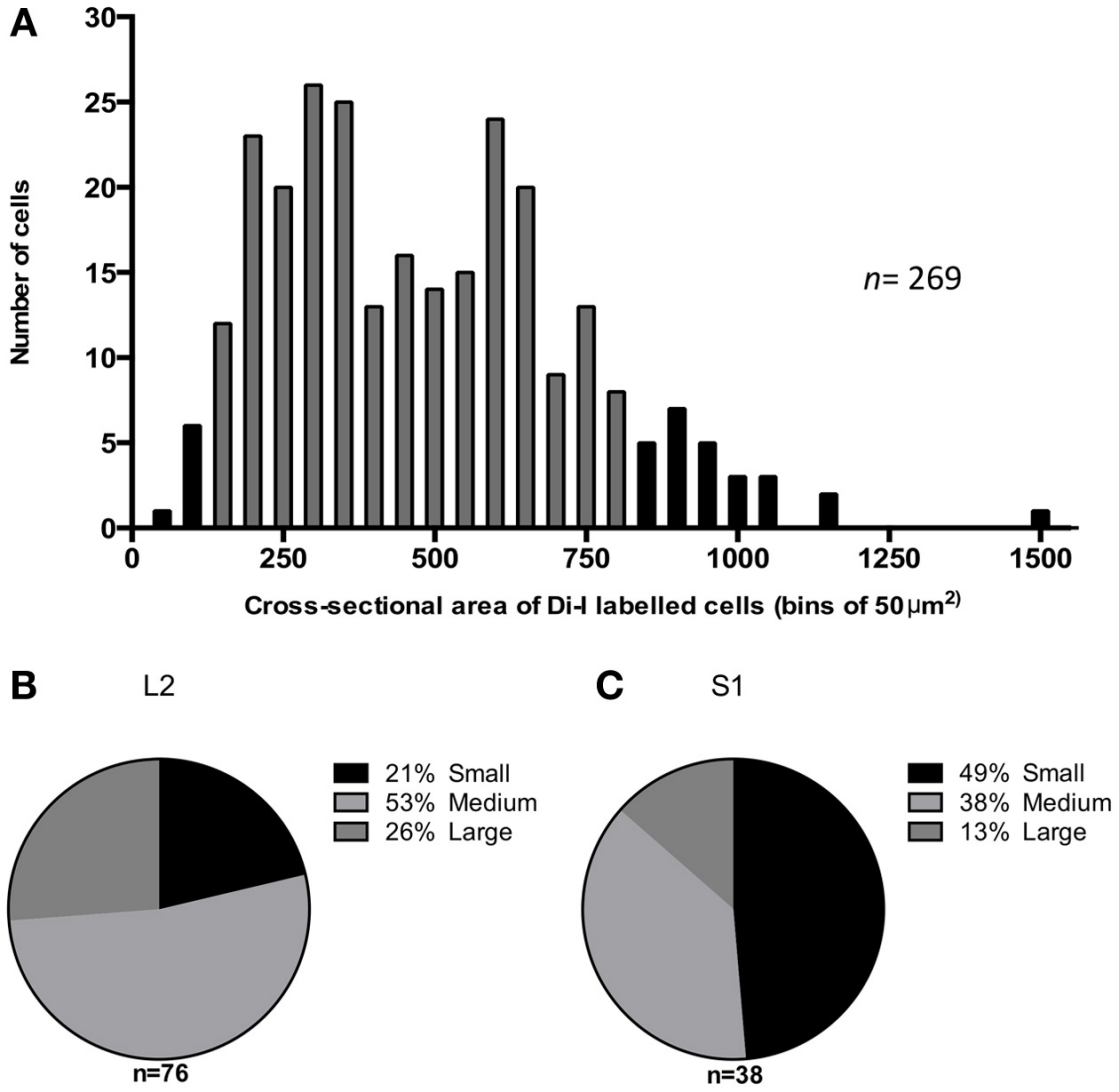
**Cross sectional area of DiI labeled cells in mouse dorsal root ganglia. (A)** Distribution of 269 labeled neurons, selected from DRGs at all spinal levels in four mice plotted as number of cells observed in each 50 μm^2^ bins of cross-sectional area. The gray bars indicate 87% of the population are between 100–800 μm^2^ **(B,C)** Proportion of DiI labeled cells that are small (<300 μm^2^), medium (300–600 μm^2^) or large (>600 μm^2^) at L2 and S1 respectively.

The majority (87%) of Di-I labeled cell bodies had a cross-sectional area between 100 and 800 μm^2^ (gray bars in Figure 3A), with the mean cross-sectional area of DiI labeled cells, 463 ± 15 μm^2^ (*n* = 269). Neurons were further classified as small (<300μm^2^), medium (300–600 μm^2^) or large (>600 μm^2^) according to their soma size. The proportion of different size-classes of cell bodies varied between the major and minor peaks of the distribution of labeled cells. As seen in Figure 3B, in the DRG at L2, 21% of the labeled cells were small, 53% medium-sized and 26% were large neurons. In comparison, the minor peak of labeled cells in the DRG at S1 (Figure 3C) had a significantly greater proportion of small neurons (49%) (*chi square, P* ≤ 0.05 2 df), 38% were medium-sized and only 13% of the labeled cells were large neurons.

### CGRP-immunoreactive innervation of the mouse uterus

**Figure 4:**
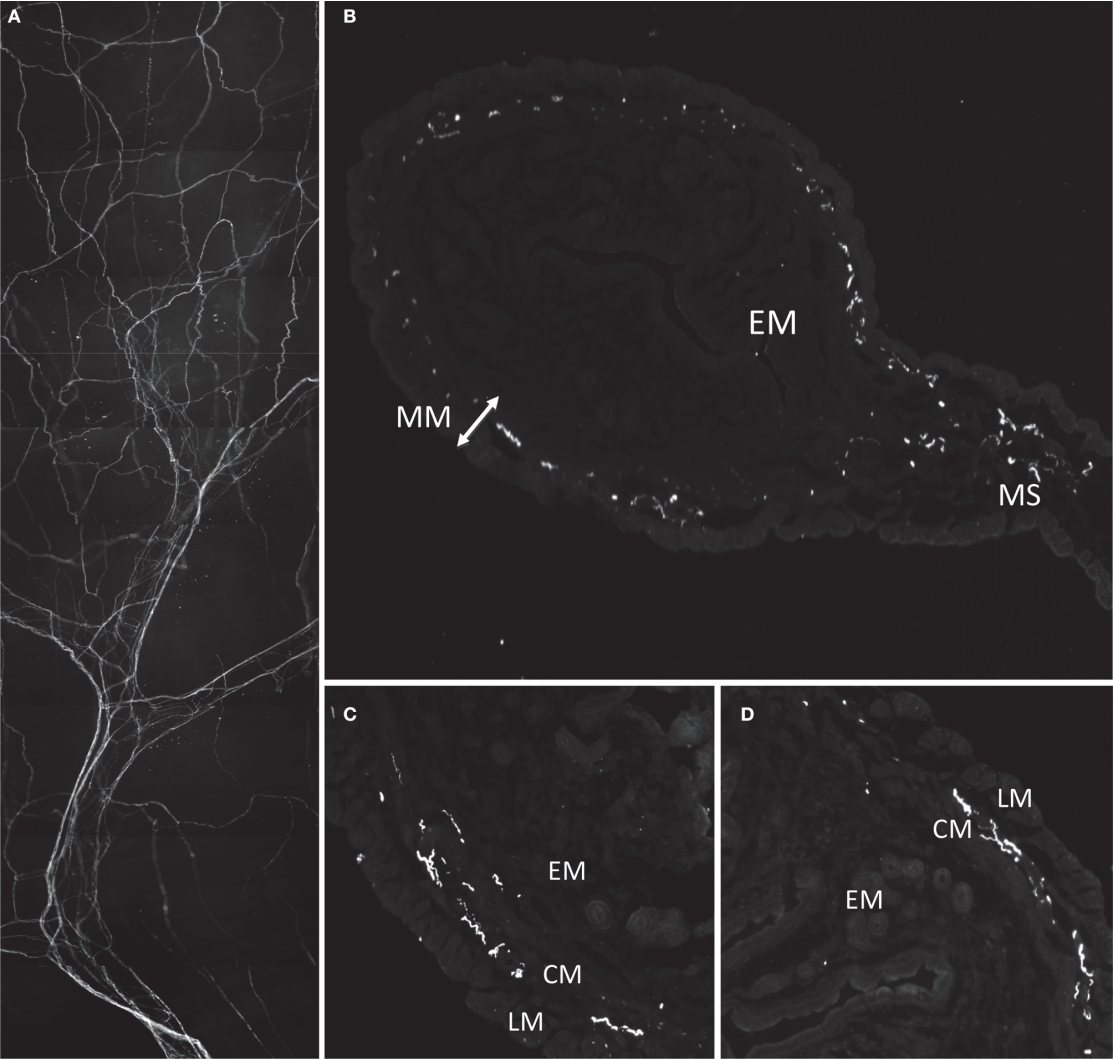
**Morphology and localization of nerve fibers in mouse uterus labeled with antibodies against CGRP. (A)** Gross morphology of nerve fiber bundles entering through mesenteric vasculature. **(B)** Cryosection of uterine horn showing localization of fibers in myometrium (MM) and mesometrium (MS) and no labeling in endometrium (EM). **(C,D)** Localization of CGRP reactive fibers predominantly localized in smooth inner circular muscle (CM) and some in outer longitudinal muscle (LM).

**Figure 5:**
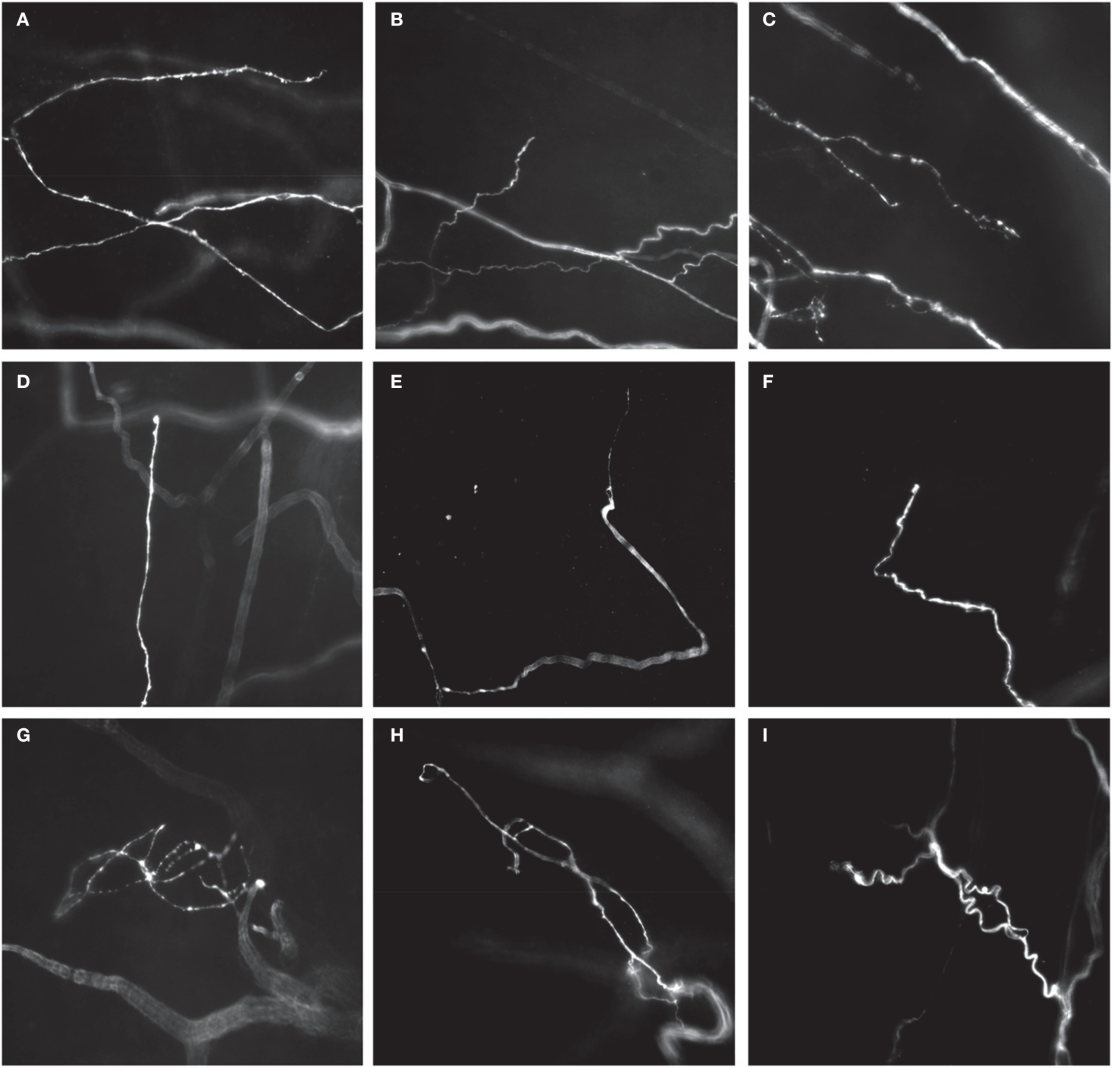
**Different types of CGRP-immunoreactive nerve endings in whole mount preparations of mouse uterine horn**. Single-type nerve endings aligned in the circumferential axis of the uterine horn, adjacent to, or within circular smooth muscle **(A–C)**, most commonly with long single axonal branches that emanate from their parent axon **(A)**, or short branches from their parent axon **(B)**. Branching-type endings were identified that consisted of multiple short branching axon terminals, with few prominent varicosities **(C)**. Some examples of single type endings are shown that have also few, if any, prominent varicosities, but were aligned in the longitudinal axis of each uterine horn **(C–F)**. **(D)** Shows a single type ending with a small bulbous terminal that lacks any complex specialization. **(E,F)** Show an axon terminal with a tapered **(E)** or hooked **(F)** endings that again lack prominent varicosities. Complex type endings were also identified that consisted of entangled nerve axons that were often found close to mesenteric vasculature **(G–I)**.

Immunoreactivity for CGRP was used to identify sensory nerves in the uterus. Most CGRP-immunoreactive axons entered the uterus at the mesometrium in bundles along with major blood vessels (Figure 4A). Thick nerve trunks entered along the mesenteric vessels and only branched three or four times as seen in Figure 4A. The axons were predominantly non-varicose and located around the large blood vessels, with minimal branching from the main/primary trunks (Figure 4A) or association to small vessels. Some axons terminated close to the major vessels (see Figures 5A–I) however the majority appeared to branch into a “honeycomb-like” mesh and terminate as simple, free endings. Sections of the uterus revealed localization of CGRP-immunoreactivity primarily in the myometrium, both in the longitudinal and circular smooth muscle layers (Figures 4B–D). Some CGRP immunoreactivity was also present in the outer regions of the endometrium (*n* = 2). There was no noticeable variation in the distribution of CGRP+ immunoreactivity between sections taken from the ovarian or cervical regions of the uterus.

Three major morphological types of CGRP immunoreactive nerve endings were identified in whole mount preparations of uterine horn. The first class was classified as a single type of free nerve ending that consisted of few, if any, discrete varicosities, and whose axons were aligned predominantly circumferentially around the uterine tube, within the circular smooth muscle (Figures 5A,B). The second type of ending was classified as a branching type (Figure 5C). These types of endings consisted of single axons that branched from their parent axon, bifurcating into at least one or two separate axons that lay parallel to each other. These endings had few, if any prominent varicosities along their axons, similar to single type endings. Interestingly, whilst single and branching type endings were found to lie circumferential to the uterine wall (Figure 5A), a number of single type endings were also found to lie parallel to longitudinal muscle (Figures 5D–F).

The third type of ending identified was classified as complex. These endings were identified by few if any varicosities along their axons, and consisted of entwined axons that wrapped around each other (Figures 5G–I), typically lying close to the mesenteric vasculature.

## Discussion

A number of novel results were obtained from this study. Perhaps most notably, we revealed that primary afferent neurons innervated the mouse uterine horn with two distinct peak densities, which lie in the thoracic and lumbar-sacral spinal segments. These sensory neurons were distributed bimodally, with one population having a peak innervation at T13-L3 and a second, smaller population at L6-S1. This bimodal distribution of sensory afferents is not unique to the uterus, as previous studies have reported similar patterns of innervation, albeit at different spinal levels, in viscera of other mammals, such as the colon and other urogenital organs (Marfut and Echtenkamp, 1991; Robinson et al., 2004; Ivanusic et al., 2013). Indeed, in larger mammals such as the pig, a bimodal distribution of spinal afferent innervation also exists in the uterus (Wasowicz et al., 1998).

Our results in the mouse uterus are similar to earlier retrograde tracing studies of the rat urogenital region, in which peak labeling was also observed at T1_12_-L_3_ and L_6_-S_1_ (Inyama et al., 1986). Although, instead of localized injections into only the uterine horns as in our study, retrograde tracing from the vagina and cervix were also included, which could explain a slightly broader distribution of labeling reaching as high up as T_12_. Other explanations could be due to inter-species variation of labeling and distribution.

Our retrograde labeling results highlight the significant overlap, or convergence, of afferent innervation of organs in the urogenital region in the mouse. The convergence of sensory information from individual pelvic structures occurs at multiple levels of the nervous system, including the dorsal root ganglia, the spinal cord and finally the brain (Malykhina, 2007). This manifests clinically in patients with chronic pelvic pain, often diagnosed with multiple conditions (Stratton and Berkley, 2010). A range of electrophysiological studies indicate the role of bi-directional cross sensitization of organs in the lower urogential tract and other abdominal visceral organs, in which the afferent irritation of one pelvic organ such as the colon adversely influences and/or sensitizes another, such as the bladder or uterus via neural interactions (Pezzone et al., 2005; Malykhina, 2007).

We were particularly interested in the relative distribution and sizes of the sensory nerve cell bodies in DRG that innervated the uterus, since recent studies have reported correlations between the size of DRG cell bodies and their specific neurochemistry in mouse intestine (Tan et al., 2008). We expected that the majority of the neurons that innervated the uterus would be small neurons that likely encode slow nociceptive reflexes, consistent with C-fibers. Our results also indicate that the majority of afferent neurons innervating the mouse uterus are small and medium-sized neurons, with a size range of <300 and 300–600 μm^2^ respectively. Our findings imply that most sensory information via the dorsal root ganglia are conveyed by small diameter unmyelinated, slow conducting C-fibers and lightly myelinated A-δ fibers. This is in accordance with well-established data of afferent innervation in other viscera such as the colon (Robinson et al., 2004). Indeed, our data correlates well with the work by Robinson et al. (2004) who showed that in the mouse colon, most of the spinal afferents that project to the colon are small CGRP-positive C-fibers (Ness and Gebhart, 1990; Robinson et al., 2004).

The second part of this study aimed to characterize calcitonin gene-related peptide (CGRP) immunoreactive nerve endings in the uterine wall, in terms of their localization and morphological features. Indeed, CGRP immunoreactivity has been observed both in the central and peripheral nervous system, the latter of which is primarily detected in afferent nerves (Inyama et al., 1986). CGRP is well known to be a potent vasodilator, playing a major role in relaxing visceral smooth muscle (Tan et al., 2008). The present study revealed substantial innervation of the mouse uterus by nerve fibers containing CGRP. Contrary to the widespread distribution we were expecting, we found CGRP fibers to be localized predominantly in the myometrium. Three major types of endings were identified. These consisted of free nerve fibers, either single or branching that aligned preferentially in the axis of either the circular or longitudinal smooth muscle. A complex class of ending was also found, that was associated with, or lay in close apposition to fine mesenteric blood vessels. This data is consistent with findings in studies of the rat uterus and indicates a functional role for CGRP in uterine smooth muscle (Shew et al., 1990). Interestingly, in rat uterus it has been described that the endometrium receives a greater innervation of CGRP positive nerve fibers than the myometrium (Ness and Gebhart, 1990; Gnanamanickan and Llewellyn-Smith, 2011). In our study, we found that the myometrium received a greater CGRP innervation than the inner endometrium. The functional significance of this difference is not clear.

In conclusion, our study has detailed the spinal level of the afferent neurons that innervate the mouse uterus as well as indicating their potential role in nociception. These findings, together with the identification of different types of nerve endings in the uterus provide a basis for exciting future studies that seek to unravel the functional role of different types of sensory endings in uterine smooth muscle.

## Significance

We have identified that, similar to the mouse colon, the mouse uterus is innervated by two distinct peaks of primary afferent neurons, that underlie the detection and transmission of noxious and innocuous stimuli from the uterine wall. We have also revealed that distinct morphological types of CGRP-positive primary afferent nerve endings innervate the smooth muscle region of the mouse uterine horn, the function of which is unclear, but may underlie the transduction of noxious and/or non-noxious stimuli.

### Conflict of interest statement

The authors declare that the research was conducted in the absence of any commercial or financial relationships that could be construed as a potential conflict of interest.
